# Ki67 expression at Kasai portoenterostomy as a prognostic factor in patients with biliary atresia

**DOI:** 10.1002/bjs5.50308

**Published:** 2020-06-16

**Authors:** D. Yoshii, Y. Inomata, Y. Komohara, K. Shimata, M. Honda, S. Hayashida, Y. Oya, H. Yamamoto, H. Yamamoto, Y. Sugawara, T. Hibi

**Affiliations:** ^1^ Department of Paediatric Surgery and Transplantation Kumamoto University Graduate School of Medical Sciences Kumamoto Japan; ^2^ Department of Cell Pathology, Graduate School of Medical Sciences Kumamoto University Kumamoto Japan; ^3^ Department of Paediatric Surgery and Transplantation Kumamoto Rosai Hospital Yatsushiro Japan

## Abstract

**Background:**

Biliary atresia is a rare paediatric biliary obliteration disease with unknown aetiology, and is the most common indication for paediatric liver transplantation (LT). However, no consensus for predicting Kasai portoenterostomy (KP) outcomes using liver histological findings exists. Ki67 is a popular biomarker for measuring and monitoring cellular proliferation.

**Methods:**

Ki67 (clone, MIB‐1) liver parenchyma expression was measured by immunohistochemical staining of samples from living donors and patients with biliary atresia to assess its value in predicting outcomes after KP.

**Results:**

Of 35 children with biliary atresia, 13 were native liver survivors (NLS), 17 were non‐NLS, and five had primary LT. The median proportion of Ki67 immunostained areas in donors and patients with biliary atresia at KP was 0·06 and 0·99 per cent respectively. Univariable analysis identified a high proportion of Ki67 areas, high Ki67 cell numbers and high Ki67‐positive/leucocyte common antigen‐positive cell numbers at KP as significant predictors of poor native liver survival after KP (hazard ratio 9·29, 3·37 and 12·17 respectively). The proportion of Ki67 areas in the non‐NLS group was significantly higher than that in the NLS group (1·29 *versus* 0·72 per cent respectively; *P* = 0·001), and then decreased at LT (0·32 per cent *versus* 1·29 per cent at KP; *P* < 0·001).

**Conclusion:**

This study has demonstrated the clinical data and time course of Ki67 expression in patients with biliary atresia. High Ki67 expression at KP may be an important predictor of native liver survival following the procedure.

## Graphical abstract

No consensus exists for predicting outcomes after Kasai portoenterostomy (KP) using liver histological findings. Ki67 (clone, MIB‐1) expression in liver parenchyma was measured by means of immunohistochemical staining of samples from five living donors and 35 patients with biliary atresia to assess its value in predicting KP outcomes. Univariable analysis identified high expression of Ki67 at the time of KP as a significant predictor of poor native liver survival after the procedure.

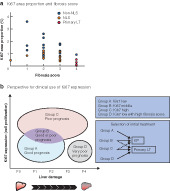

Ki67 a potential marker for liver survival after Kasai portoenterostomy

## Abstract (es)

**Antecedentes:**

La atresia biliar (*biliary atresia*, BA) es una enfermedad pediátrica rara que consiste en una obstrucción biliar de etiología desconocida, y es la indicación pediátrica más frecuente de trasplante hepático (*liver transplantation*, LT). Sin embargo, no existe consenso para predecir los resultados de la portoenterostomía de Kasai (Kasai *portoenterostomy*, KP) en base a los hallazgos histológicos hepáticos. El Ki67 es un biomarcador conocido para medir y controlar la proliferación celular.

**Métodos:**

Se midieron los niveles de expresión del parénquima hepático de Ki67 (clon, MIB‐1) por tinción inmunohistoquímica de las muestras de cinco donantes vivos y 35 pacientes con BA, para evaluar su valor predictivo de los resultados de la KP.

**Resultados:**

Los pacientes con BA incluían 13 sobrevivientes con hígado nativo (*native liver survivors*, NLS), 17 no NLS y 5 pacientes que se sometieron inicialmente a LT. La proporción media de las áreas de expresión de Ki67 en donantes y pacientes con BA en KP fue de 0,06% y 0,99%, respectivamente. El análisis univariado identificó una alta proporción de áreas de Ki67, un alto número de células Ki67, un alto número de células Ki67 positivas (+)/leucocitos (LCA/CD45) + en KP como predictores significativos de una peor supervivencia del hígado nativo después de KP (cociente de riesgos instantáneos, *hazard ratio*, HR 9,29, 3,37 y 12,17, respectivamente). La proporción de las áreas Ki67 fueron significativamente superiores en los pacientes sin NLS que en los pacientes con NLS (*P* = 0,001). Entre los pacientes sin hígado nativo, los niveles de Ki67 disminuyeron posteriormente de acuerdo con la presencia de una lesión hepática irreparable, tales como son los hígados con BA en LT (en KP *versus* en LT = 1,29% *versus* 0.32%; *P* < 0,001).

**Conclusión:**

Demostramos los datos clínicos y la evolución temporal de la expresión de Ki67 en los pacientes con BA. El alto nivel de expresión de Ki67 en KP puede ser un predictor importante para la supervivencia del hígado nativo después de KP.

## Introduction

Biliary atresia is a rare paediatric disease characterized by an obliterative cholangiopathy of unknown pathogenesis^1^. Kasai portoenterostomy (KP) is usually performed during early infancy, but subsequent liver transplantation (LT) is necessary in failed cases. Biliary atresia is the most common indication for paediatric LT, and accounts for up to 50 per cent of paediatric LTs globally^1^. Although studies have tested histological variables to predict outcomes following KP, the results have been contradictory and no consensus exists on the most appropriate histological finding, such as the number or size of biliary ductules in the remnant bile duct^2^, ^3^, ^4^, ^5^, the presence of giant cell transformation^6^, ^7^, ^8^, the degree of bile duct inflammation^8^, ^9^, ^10^, ^11^, or the amount of liver fibrosis and ductular reaction^6^, ^12^, ^13^, ^14^, ^15^. Prediction of outcome after KP would allow patients with a poor prognosis to be sufficiently prepared for upcoming LT. In addition, knowledge of the causes of poor prognosis after KP would enable researchers to develop new treatments for patients with biliary atresia. Thus, finding a good prognostic biomarker is important.

Ki67 has been a popular biomarker for measuring and monitoring tumour proliferation for many years, especially in breast cancer^16^. Expression of Ki67 varies throughout the cell cycle, reaching a peak during mitosis and being absent during the G0 stage of the cell cycle^16^. The present authors hypothesized that advanced liver damage at KP might result in a poor outcome. In view of the regeneration potential of the liver, cellular turnover is expected to be faster than normal when the liver is damaged^17^, ^18^, ^19^, ^20^. Thus, Ki67 may be an objective biomarker with which to predict prognosis after KP. Only one report^21^ has been published on the association between Ki67 expression in bile duct cells and clinical outcome in patients with biliary atresia, and there appear to be no reports on the relationship between Ki67 expression in other liver cells and clinical outcome in these patients. There is also a lack of studies reporting on the time course of Ki67 expression according to liver functioning. The aim of this study was to assess Ki67 expression as a histological, prognostic and liver functional marker after KP in patients with biliary atresia.

## Methods

This retrospective study used data from patients with biliary atresia who had undergone KP and/or LT in Kumamoto University Hospital, Kumamoto, Japan, and from living LT donors between January 2005 and December 2016. The living LT donors are described as ‘donors’ and those who had primary LT are described as ‘primary patients’ in this article.

All clinical data were obtained from electronic medical charts. Liver specimens collected from the periphery of native livers by wedge resection during surgery were used for liver histological examination in clinical practice; in the present study, the remaining specimens were used for research purposes. The fibrosis score of liver specimens was determined using the new Inuyama classification^22^: F0, no portal fibrosis; F1, fibrous portal expansion; F2, bridging fibrosis; F3, bridging fibrosis with lobular distortion; and F4, cirrhosis.

The institutional review board at Kumamoto University Hospital approved this retrospective study (number 371). Written informed consent was obtained from each patient (if they could understand the study aim) and their parents, or only from parents (when patients were too young to understand the aim).

### Immunohistochemical staining

Specimens were fixed at room temperature for at least 72 h in 10 per cent formalin (Wako Pure Chemical Industries, Osaka, Japan) and embedded in paraffin (Sakura Finetek, Tokyo, Japan). Specimens were then sectioned at 3 μm. The immunohistochemical (IHC) staining protocol has been described previously^23^. Sections were immersed in EDTA solution (pH 8·0) for retrieval of the antigens Ki67, cytokeratin (CK) 19 and cluster of differentiation (CD) 163, and in citrate buffer (pH 6·0) (LSI Medience, Tokyo, Japan) for retrieval of hepatocyte paraffin (HepPar) 1, leucocyte common antigen (LCA/CD45) and α‐smooth muscle actin (αSMA). Samples were heated in a pressure cooker (Agilent, Santa Clara, California, USA) at 125°C for 5 min.

The primary antibodies and concentrations used were: mouse anti‐Ki67 (clone MIB‐1) at 1 : 100 (Agilent), mouse anti‐HepPar1 at 1 : 50 (Agilent), mouse anti‐LCA at 1 : 200 (Agilent), mouse anti‐αSMA at 1 : 100 (Agilent), rabbit anti‐CK19 at 1 : 1000 (Abcam, Cambridge, UK) and mouse anti‐CD163 (clone 10D6) at 1 : 300 (Novocastra, Newcastle, UK). The samples were incubated with horseradish peroxidase‐labelled goat antimouse or rabbit secondary antibodies (Nichirei Biosciences, Tokyo, Japan). Immunoreactions were visualized using a diaminobenzidine (DAB) substrate kit (Nichirei Biosciences). For double‐immunostaining, sections visualized with DAB in the first immunostaining were treated twice for 5 min by microwaving them in citrate buffer, and the second immunostaining was then performed, with the reaction visualized using HistoGreen (LINARIS Biologische Produkte, Dossenheim, Germany). Double‐IHC staining was performed using three pairs of markers with anti‐Ki67, including anti‐HepPar1 (for hepatocytes), anti‐LCA (for inflammatory cells, including Kupffer cells) and anti‐αSMA (for hepatic stellate cells) antibodies.

### Image processing and cell counting

To quantify the proportion of immunostained areas per field (described in this study as the ‘area proportion’), using ImageJ 1.46 (National Institutes of Health, Bethesda, Maryland, USA), three non‐overlapping, randomly selected areas (except portal areas) were viewed for each patient; for CK19‐immunostained sections, the portal areas were included. Immunostained sections were photographed with a microscope (BX51; Olympus, Tokyo, Japan) at 40× magnification for CK19 and 200× magnification for the others. Images of the immunostained sections were converted into 8‐bit greyscale images that had only colour intensity. Threshold values for positive signals of the selected images were adjusted (from 0 to 255), and were set at 100 for Ki67 and CK19, and 120 for CD163. This was because compensated images were more reflective of original images. The area proportion was then calculated. To quantify immunostaining by cell counting, six non‐overlapping areas (except portal areas) were selected randomly for the analyses. Immunostained sections were photographed at 400× magnification. Two pathologists, blinded to patient information, evaluated all sections.

### Statistical analysis

All data are presented as median (range) values. The Mann–Whitney *U* test was used for comparisons between groups, and the Wilcoxon test for comparisons between two paired groups. To estimate the predictors of native liver survival after KP, receiver operating characteristic (ROC) curves were generated for each clinical parameter generally used in clinical practice and for Ki67 values, and the areas under the ROC curves (AUROCs) were compared. Cut‐off values were determined by the Youden index based on the ROC curves, and patients with biliary atresia were divided into two groups on the basis of those values. Kaplan–Meier curves were generated for native liver survival rates, and groups were compared with the log rank test. Spearman's rank test was used to study associations between two variables. All tests were two‐sided, and *P* < 0·050 was considered statistically significant. Statistical analyses were performed with GraphPad Prism® 7 (GraphPad Software, San Diego, California, USA).

## Results

At October 2018, of 35 children with biliary atresia, 13 were native liver survivors (NLS) and 17 were non‐NLS; these 17 patients underwent both KP and the subsequent LT at the authors' institution. The remaining five patients were primary patients.

*Table* *1* presents basic clinical and biochemical data for patients with biliary atresia, excluding primary patients. Nine patients were boys and 21 were girls. Their median gestational age, birthweight and age at KP were 39 (31–41) weeks, 2877 (940–4250) g and 69 (27–143) days respectively. Twenty‐nine patients had type III biliary obstruction^1^, and one patient had type I. The jaundice clearance rate was 53 per cent (16 of 30). *Table* *2* presents basic clinical and biochemical data for patients with biliary atresia in the NLS, non‐NLS (at KP), primary patient and non‐NLS (at LT) groups.

**Table 1:** Basic clinical and biochemical data for patients with biliary atresia (excluding those who had primary liver transplantation)

	No. of patients (*n* = 30)
**NLS** : **non‐NLS**	13 : 17
**Gestational age (weeks)** *	39 (31–41)
**Sex ratio (M** : **F)**	9 : 21
**Birthweight (g)** *	2877 (940–4250)
**Age at KP (days)** *	69 (27–143)
**Total bilirubin (mg/dl)** *	9·1 (4·8–11·6)
**Direct bilirubin (mg/dl)** *	6·1 (2·6–8·0)
**γ‐GTP (units/l)** *	376 (110–1896)
**Total bile acids (μmol/l)** *	121·9 (68·2–327·4)
**AST (units/l)** *	155 (54–338)
**ALT (units/l)** *	90 (21–300)
**Platelets (× 10** ^**3**^ **/μl)** *	437 (171–763)
**PT‐INR** *	1·03 (0·82–1·44)
**APRI** *	1·17 (0·29–4·17)
**Type of biliary atresia**	
I	1
II	0
III	29
**Duration of surgery (min)** *	282 (216–397)
**Blood loss (g)** *	23·5 (0–80)
**Fibrosis score** *	2 (0–4)
**Jaundice clearance achieved**	16

* Values are median (range). NLS, native liver survivor; KP, Kasai portoenterostomy; GTP, glutamyl transferase; AST, aspartate aminotransferase; ALT, alanine aminotransferase; PT‐INR, prothrombin time international normalized ratio; APRI, AST to platelet ratio index.

**Table 2:** Basic clinical and biochemical data for patients with biliary atresia according to group

	NLS (*n* = 13)	Non‐NLS (at KP) (*n* = 17)	Primary patients (*n* = 5)	Non‐NLS (at LT) (*n* = 17)
Age at KP (days)*	60 (38–88)	69 (27–143)	–	69 (27–143)
Age at LT (days)*	–	–	165 (130–216)	209 (123–622)
Gestational age (weeks)*	39 (31–41)	39 (31–41)	35 (29–41)	39 (31–41)
Sex ratio (M : F)	4 : 9	5 : 12	1 : 4	5 : 12
Birthweight (g)*	2876 (2124–3374)	2982 (940–4250)	2608 (1200–3272)	2982 (940–4250)
Total bilirubin (mg/dl)*	9·3 (5·6–11·6)	9·0 (4·8–10·3)	19·7 (10·1–32·6)	13·1 (0·9–42·7)
Direct bilirubin (mg/dl)*	6·3 (2·6–8·0)	6·0 (3·1–6·8)	11 (6·4–15·2)	9·1 (0·4–28)
γ‐GTP (units/l)*	580 (139–1896)	371 (110–1025)	99 (19–171)	170 (24–513)
Total bile acids (μmol/l)*	137·2 (93·8–327·4)	114·4 (68·2–225·1)	110·3 (23·1–215·6)	148·5 (26·5–310·2)
AST (units/l)*	149 (56–338)	162 (54–303)	342 (39–585)	171 (69–1221)
ALT (units/l)*	101 (21–283)	89 (26–300)	174 (13–318)	119 (30–714)
Platelets (× 10^3^/μl)*	490 (274–763)	327 (171–721)	190 (1·7–370)	167 (59–354)
PT‐INR*	1·03 (0·82–1·31)	1·07 (0·88–1·44)	1·37 (1·23–2·52)	1·37 (0·91–1·79)
APRI*	0·82 (0·32–2·67)	1·27 (0·29–4·17)	4·86 (2·77–67·47)	3·90 (0·57–16·1)
Total protein (g/dl)*	5·6 (4·9–6·4)	5·8 (4·3–6·9)	6·0 (4·2–8·4)	5·8 (4·2–7·4)
Cholinesterase (units/l)*	269 (141–359)	272 (167–397)	96 (51–383)	108 (61–212)
Total cholesterol (mg/dl)*	182 (122–448)	181 (96–292)	149 (78–270)	150 (75–215)

* Values are median (range). NLS, native liver survivor; LT, liver transplantation; KP, Kasai portoenterostomy; GTP, glutamyl transferase; AST, aspartate aminotransferase; ALT, alanine aminotransferase; PT‐INR, prothrombin time international normalized ratio; APRI, AST to platelet ratio index.

### Ki67 immunohistochemical staining in liver specimens

**Figure 1:**
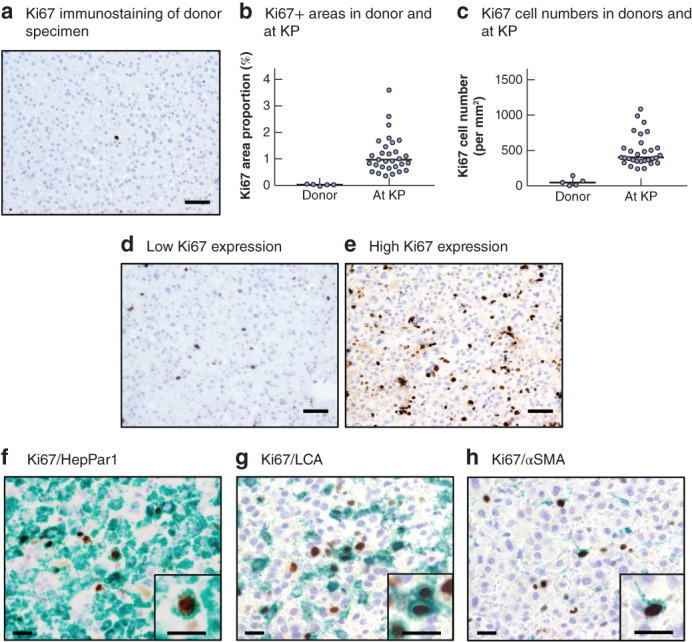
Representative Ki67 expression in liver specimens from living donors and patients with biliary atresia **a** Representative Ki67 immunohistochemical (IHC) staining from donor liver specimen. Scale bar, 50 μm. **b** Dot plot of Ki67‐positive (+) areas of donor and patients with biliary atresia at Kasai portoenterostomy (KP). **c** Dot plot of Ki67 cell numbers in donors and patients with biliary atresia at KP. Horizontal bars indicate median values. **d,e** Representative low and high Ki67 IHC expression in liver of patients with biliary atresia. Scale bar, 50 μm. **f–h** Representative IHC staining of Ki67+/hepatocyte paraffin (HepPar) 1+, Ki67+/leucocyte common antigen (LCA)+ and Ki67+/α‐smooth muscle actin (αSMA)+ cells in liver from patients with biliary atresia. Scale bars, 20 μm.

To investigate Ki67 expression in liver specimens, IHC staining was performed using five donor livers and livers from patients with biliary atresia. There were few Ki67‐positive (+) cells in donor livers (*Fig*. *1a*). In donors, the median Ki67 area proportion and cell numbers were 0·06 (0·01–0·10) per cent and 47 (0–146) per mm^2^ respectively (*Fig*. *1b,c*). The respective median values for the 30 patients with biliary atresia (excluding primary patients) were 0·99 (0·40–3·60) per cent and 403 (244–1097) per mm^2^ respectively (*Fig*. *1b,c*). Ki67 expression varied among patients with biliary atresia (*Fig*. *1d*).

The liver has many types of cell, including hepatocytes, Kupffer cells, hepatic stellate cells and cholangiocytes. To identify which cells in patients with biliary atresia expressed Ki67, double IHC staining was performed. This showed that cells expressing Ki67 included HepPar1, LCA and αSMA cells (*Fig*. *1e*).

### Factors associated with native liver survival after Kasai portoenterostomy

Among the AUROCs of clinical parameters and Ki67 values, large values were found for Ki67 area proportion (AUROC 0·83, *P* = 0·002), Ki67 cell number (AUROC 0·73, *P* = 0·036) and platelet count (AUROC 0·74, *P* = 0·028) (*Table* *3*; *Fig. S1a–c*, supporting information).

**Table 3:** Receiver operating characteristic (ROC) analyses for estimating prognostic factors after Kasai portoenterostomy

	AUROC	*P*	Cut‐off value
Ki67 area proportion (%)	0·83 (0·69, 0·98)	0·002	0·77
Ki67 cell number (per mm^2^)	0·73 (0·54, 0·91)	0·036	382
Ki67+/LCA+ cell number (per mm^2^)	0·67 (0·46, 0·89)	0·126	117
Ki67+/HepPar1+ cell number (per mm^2^)	0·62 (0·40, 0·83)	0·297	153
Ki67+/αSMA+ cell number (per mm^2^)	0·60 (0·37, 0·83)	0·393	88
Age at KP (days)	0·51 (0·30, 0·72)	0·933	60
Total bilirubin (mg/dl)	0·53 (0·32, 0·74)	0·769	7·95
Direct bilirubin (mg/dl)	0·54 (0·32, 0·76)	0·691	6·25
γ‐GTP (units/l)	0·59 (0·36, 0·82)	0·414	700·5
Total bile acids (μmol/l)	0·69 (0·49, 0·88)	0·082	124·8
AST (units/l)	0·51 (0·30, 0·73)	0·917	317
ALT (units/l)	0·51 (0·29, 0·72)	0·950	118
Platelets (× 10^3^/μl)	0·74 (0·56, 0·92)	0·028	340
PT‐INR	0·65 (0·44, 0·85)	0·174	1·07
APRI	0·65 (0·44, 0·85)	0·174	1·77
Duration of surgery (min)	0·69 (0·47, 0·92)	0·075	299
Blood loss (g)	0·50 (0·28, 0·72)	0·983	27·5
Fibrosis score	0·52 (0·31, 0·73)	0·867	2

Values in parentheses are 95 per cent confidence intervals. AUROC, area under the receiver operating characteristic (ROC) curve; +, positive; LCA, leucocyte common antigen; HepPar, hepatocyte paraffin; αSMA, α‐smooth muscle actin; KP, Kasai portoenterostomy; GTP, glutamyl transferase; AST, aspartate aminotransferase; ALT, alanine aminotransferase; PT‐INR, prothrombin time international normalized ratio; APRI, AST to platelet ratio index.

Univariable analysis was performed to explore the risk factors associated with native liver survival after KP. High Ki67 area proportion, high Ki67 cell number, high Ki67+/LCA+ cell number, low platelet count, high PT‐INR and low duration of surgery were significant predictors of poor native liver survival (*Table* *4*; *Fig*. *S1d–f*, supporting information). No association was found between survival and other clinical parameters, Ki67+/HepPar1+ cell number or Ki67+/αSMA+ cell number. Livers with high Ki67 area proportions had a significantly worse native liver survival rate than those with low Ki67 area proportions (hazard ratio (HR) 9·29, 95 per cent c.i. 3·47 to 24·91; *P* = 0·008) (*Fig. S1d*, supporting information). Livers with a high proportion of Ki67+/LCA+ cells had a significantly worse native liver survival rate than those with a low proportion (HR 12·17, 3·92 to 37·78; *P* = 0·002) (*Fig. S1f*, supporting information).

**Table 4:** Univariable analysis of risk factors associated with native liver survival after Kasai portoenterostomy

	Hazard ratio	*P* *
Ki67 area proportion ≥ 0·77%	9·29 (3·47, 24·91)	0·008
Ki67 cell number ≥ 382/mm^2^	3·37 (1·28, 8·82)	0·040
Ki67+/LCA+ cell number ≥ 117/mm^2^	12·17 (3·92, 37·78)	0·002
Ki67+/HepPar1+ cell number ≥ 153/mm^2^	1·65 (0·58, 4·74)	0·345
Ki67+/αSMA+ cell number ≥ 88/mm^2^	2·32 (0·84, 6·40)	0·111
Age at KP ≥ 61 days	1·23 (0·48, 3·20)	0·668
Total bilirubin < 7·95 mg/dl	1·71 (0·56, 5·19)	0·282
Direct bilirubin < 6·25 mg/dl	1·49 (0·57, 3·87)	0·430
γ‐GTP < 700·5 units/l	3·24 (1·15, 9·12)	0·100
Total bile acids < 124·8 μmol/l	2·55 (0·99, 6·61)	0·065
AST < 317 units/l	3·06 (0·60, 15·50)	0·192
ALT ≥ 118 units/l	1·41 (0·51, 3·86)	0·483
Platelets < 340 × 10^3^/μl	3·01 (1·03, 8·76)	0·015
PT‐INR ≥ 1·07	3·04 (1·04, 8·87)	0·015
APRI ≥ 1·77	2·37 (0·81, 6·88)	0·062
Duration of surgery < 299 min	6·92 (2·67, 17·93)	0·002
Blood loss ≥ 27·5 g	1·72 (0·56, 5·21)	0·279
Fibrosis score ≥ 2	1·11 (0·42, 2·96)	0·825

+, Positive; LCA, leucocyte common antigen; HepPar, hepatocyte paraffin; αSMA, α‐smooth muscle actin; KP, Kasai portoenterostomy; GTP, glutamyl transferase; AST, aspartate aminotransferase; ALT, alanine aminotransferase; PT‐INR, prothrombin time international normalized ratio; APRI, AST to platelet ratio index.

* Log rank test.

### Comparison of Ki67 expression in native liver survivors and non‐survivors

**Figure 2:**
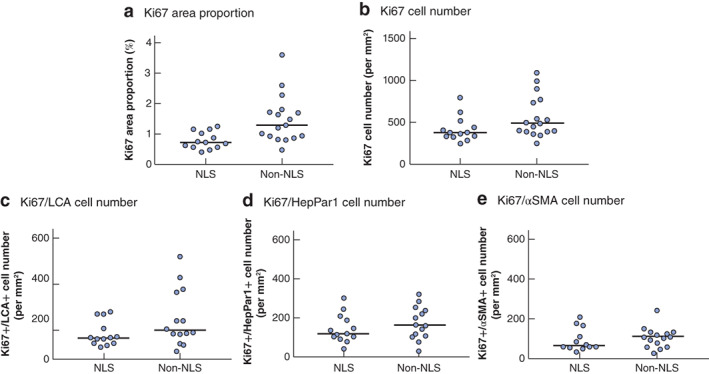
Dot plots of Ki67 expression and cell numbers in native liver survivors and non‐native liver survivors Median values for **a** Ki67 area proportions, **b** Ki67 cell number, **c** Ki67‐positive (+)/leucoctye common antigen (LCA)+ cell numbers, **d** Ki67+/hepatocyte paraffin (HepPar) 1+ cell numbers, and **e** Ki67+/α‐smooth muscle actin (αSMA)+ cell numbers in native liver survivors (NLS) and non‐native liver survivors (non‐NLS). **a** *P* = 0·001, **b** *P* = 0·036, **c** *P* = 0·130, **d** *P* = 0·307, **e** *P* = 0·404 (Mann–Whitney *U* test).

The Ki67 area proportion, identified as a significant predictor of poor native liver survival in univariable analysis, was compared in NLS and non‐NLS groups. The median Ki67 area proportion was significantly higher in the non‐NLS group (1·29 per cent *versus* 0·72 per cent in the NLS group; *P* = 0·001) (*Fig*. *2a*). The median Ki67 cell number was also significantly higher in the non‐NLS group (489 *versus* 374 cells/mm^2^ respectively; *P* = 0·036) (*Fig*. *2b*).

Numbers of Ki67+/LCA+ cells, also identified in univariable analysis as a significant prognostic predictor in livers with biliary atresia, were then compared. Numbers of Ki67+/LCA+ cells were higher in the non‐NLS than in the NLS group, but the difference was not significant (*P* = 0·130) (*Fig*. *2c*). Numbers of Ki67+/HepPar1+ and Ki67+/αSMA+ cells were also higher in the non‐NLS group, but again the differences were not significant (*P* = 0·307 and *P* = 0·404 respectively) (*Fig*. *2d,e*).

### Change in Ki67 expression between Kasai portoenterostomy and liver transplantation

**Figure 3:**
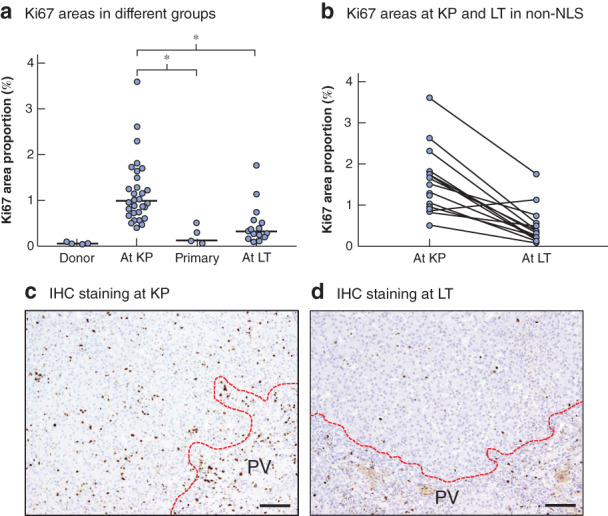
Ki67 expression changes in liver from patients with biliary atresia **a** Comparison of Ki67 areas in liver specimens of 30 patients with biliary atresia at the time at Kasai portoenterostomy (KP), five patients who had primary liver transplantation (LT) and 17 patients with biliary atresia at the time at LT. Values for five living LT donors are shown as a control. Horizontal bars indicate median values. **b** Time course of the proportion of Ki67 areas in liver specimens at KP and LT in non‐native liver survivors with biliary atresia. **c,d** Representative immunohistochemical (IHC) staining showing Ki67 expression in liver specimens at KP and LT in the same patient with biliary atresia. PV, portal vein area. Scale bars, 200 μm. **a** *P* < 0·001 (Mann–Whitney *U* test).

To explore the association between Ki67 expression and the time course after KP, Ki67 expression was examined in donor livers (as normal livers) and livers with biliary atresia (*Fig*. *3a*), obtained at KP (30 livers), primary LT (5) or LT (17). LT specimens were obtained at the time of LT from patients with biliary atresia who had undergone KP in the authors' institution. Median patient age at LT was 209 (123–622) days (*Table* *2*). The median Ki67 area proportion in the KP group was higher than that in the donor group (*Fig*. *3a*). The median Ki67 area proportion in the primary group was significantly lower than that in the KP group (0·12 *versus* 0·99 per cent respectively; *P* < 0·001). The Ki67 area proportion in the LT group was significantly lower than that in the KP group (0·32 *versus* 0·99 per cent respectively; *P* < 0·001) (*Fig*. *3a*).

To ascertain the time course of Ki67 expression in detail, Ki67 expression in livers of patients in the non‐NLS group was compared between the times of KP and LT. Almost all of the Ki67 area proportions decreased with time, and median Ki67 expression at LT was significantly lower than that at KP (0·32 *versus* 1·29 per cent respectively; *P* < 0·001, Wilcoxon test) (*Fig*. *3b*). *Fig*. *3c,d* shows representative images that demonstrate the time course of Ki67 expression at KP and LT in the same patient.

**Figure 4:**
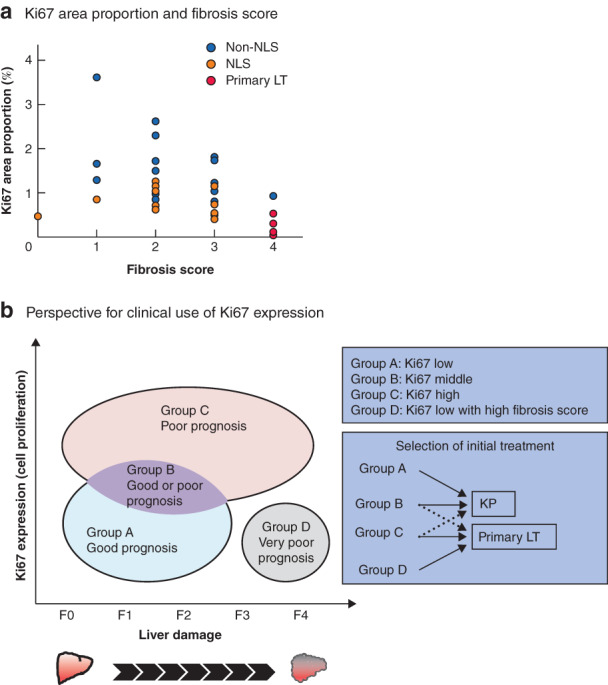
Ki67 area proportion, fibrosis score, and perspective for the clinical use of Ki67 expression **a** Dot plot of Ki67 area proportion for each fibrosis score for all patients with biliary atresia: native liver survivors (NLS), non‐NLS and primary liver transplantation (LT) groups. **b** Schematic diagram providing a perspective for the clinical use of Ki67 expression. Patients with biliary atresia are distributed by Ki67 area proportion for each fibrosis score (F0–F4). The grouping corresponds with their prognosis and selects their first treatment, Kasai portoenterostomy (KP) or primary LT.

To investigate the association between Ki67 expression and liver function, a dot plot was generated to demonstrate Ki67 expression and the fibrosis score for all patients with biliary atresia. Patients in the NLS group appeared mainly in the left lower part of the plot, those in the non‐NLS group in the upper part, and the primary patients in the right lower part (*Fig*. *4a*).

## Discussion

This study found that Ki67 expression in liver parenchyma of patients with biliary atresia might be used as a biomarker for predicting native liver survival after KP, and that high Ki67 expression and Ki67+/LCA+ cell numbers at the time of KP were associated with poor clinical prognosis. In addition, Ki67 expression in livers with biliary atresia decreased as liver function deteriorated.

Immunohistochemical detection of the Ki67 antigen has been used for many years to assess cancer proliferation^16^. However, few reports have focused on Ki67 as a biomarker in non‐tumour diseases such as biliary atresia. Funaki and colleagues^24^ demonstrated that the Ki67 labelling index in the biliary ducts of livers with biliary atresia was higher than that in normal control livers, but the differences were not statistically significant. Kinugasa and co‐workers^21^ found that the MIB‐1 index in biliary cells did not differ between patients with biliary atresia and poor bile drainage and those with good bile drainage. These results suggested that assessment of Ki67 expression in bile ducts could not be used for prognosis in biliary atresia. However, Hossain *et al*.^7^ reported that the expression in hepatocytes of proliferating cell nuclear antigen, a marker of cellular proliferation, was closely related to the prognosis of patients with biliary atresia. Therefore, measuring Ki67 alone in liver parenchyma may help obtain an accurate prognosis for patients with biliary atresia. This result is reasonable, because an increase in Ki67 cell numbers occurs as a result of active cellular reactions that may be the response to liver injury^17^, ^20^. Patients with a high Ki67 area proportion may have more severe liver damage than those with a low Ki67 area proportion. IHC assessment of the Ki67 labelling index is the method used most widely to determine cell proliferation; however, standardization of Ki67 staining and cell counting is problematic, and interlaboratory reproducibility is variable^25^.

Few reports have been published on the preoperative identification of patients with biliary atresia and a poor prognosis following KP. Azarow and colleagues^10^ found that the presence of syncytial giant cells, lobular inflammation, focal necrosis, bridging necrosis and cholangitis in preoperative liver biopsy samples was associated with failure of KP, consistent with the present authors' findings that a high number of Ki67+/LCA+ cells at KP was associated with poor prognosis. LCA is a marker of inflammatory cells, including Kupffer cells. Therefore, a high accumulation of inflammatory cells in livers with biliary atresia may be an important factor for assessing prognosis after KP. Liver fibrosis has also been associated with the prognosis of patients with biliary atresia after KP^12^, ^15^. High perisinusoidal deposition of type I collagen at KP was associated with poor progression after KP^26^. A high level of αSMA expression was also significantly associated with poor KP outcomes^27^, ^28^, ^29^. Although the number of Ki67+/αSMA+ cells in the non‐NLS group was higher than that in the NLS group in the present study, this did not appear to have any association with native liver survival. CD163 is a specific marker of activated macrophages associated with liver fibrosis^30^, ^31^; however, little evidence was found in the present study for an association with Ki67 (*Fig*. *S2*, supporting information). This may indicate that the activity of inflammatory cells may be more important for the prognosis of patients with biliary atresia than that of liver stellate cells. One report^32^ demonstrated that pharmacological inhibition and antibody neutralization of serum matrix metalloproteinase (MMP) 7 suppressed the experimental biliary atresia phenotype in neonatal mice infected by rotavirus. MMP acts on proinflammatory cytokines, chemokines and other proteins to regulate inflammation^33^. Thus, inhibition of inflammation may contribute to improving the prognosis of patients with biliary atresia before or after KP as a future therapeutic possibility.

The Ki67 expression changes between KP and LT were an intriguing finding. Ki67 expression increased at KP and decreased at LT to the same level as that in donors, and Ki67 expression decreased from KP to that at LT among the same patients in the non‐NLS group. Ki67 expression in the primary patients was significantly lower than that in patients with biliary atresia in the KP group. These results suggest that the change in Ki67 expression was due to liver function changes and/or liver damage in patients with biliary atresia.

The combination of Ki67 expression and fibrosis score may be helpful to separate patients with biliary atresia into four types (groups A–D) that predict different prognoses (*Fig*. *4b*), and initial treatment (KP or primary LT) may be selected according to this grouping. At present, no consensus exists on primary LT indications. Thus, if liver biopsy is used before intraoperative cholangiography to determine Ki67 expression and the fibrosis score, the values of these two histological parameters may be used as an indicator for primary LT (for example, extremely high Ki67 expression or extremely low Ki67 expression with a high fibrosis score).

A limitation of this study is the small number of patients included. In addition, the age of control patients was different to that of patients with biliary atresia, and there was no age‐matching.

As KP procedures have improved, the clinical outcome of patients with biliary atresia has also improved; however, the jaundice clearance rate is only around 60 per cent^1^, ^34^. Therefore, many patients already have irreversible liver changes at the time of KP. High‐quality biomarkers that can assess success before surgery are needed. This study compared liver histological findings at the time of surgery, and the findings need to be verified using preoperative biopsy samples. The use of biopsy samples to assess outcomes before surgery would enable the selection of patients for primary LT, although for patients with low expression of Ki67 it would be difficult to decide on the indication for KP or primary LT. This approach should be validated in future studies with more patients.

## Supporting information


**Appendix S1**: Supporting information

## References

[1] Hartley JL , Davenport M , Kelly DA . Biliary atresia. Lancet 2009; 374: 1704–1713. 19914515 10.1016/S0140-6736(09)60946-6

[2] Kasai M. Treatment of biliary atresia with special reference to hepatic porto‐enterostomy and its modifications. Prog Pediatr Surg 1974; 6: 5–52. 4596366

[3] Lawrence D , Howard ER , Tzannatos C , Mowat AP . Hepatic portoenterostomy for biliary atresia. A comparative study of histology and prognosis after surgery. Arch Dis Child 1981; 56: 460–463. 7259277 10.1136/adc.56.6.460 PMC1627475

[4] Mirza Q , Kvist N , Petersen BL . Histologic features of the portal plate in extrahepatic biliary atresia and their impact on prognosis – a Danish study. J Pediatr Surg 2009; 44: 1344–1348. 19573659 10.1016/j.jpedsurg.2008.11.054

[5] Miyano T , Suruga K , Tsuchiya H , Suda K . A histopathological study of the remnant of extrahepatic bile duct in so‐called uncorrectable biliary atresia. J Pediatr Surg 1977; 12: 19–25. 833710 10.1016/0022-3468(77)90291-3

[6] Vazquez‐Estevez J , Stewart B , Shikes RH , Hall RJ , Lilly JR . Biliary atresia: early determination of prognosis. J Pediatr Surg 1989; 24: 48–51. 2723994 10.1016/s0022-3468(89)80300-8

[7] Hossain M , Murahashi O , Ando H , Iio K , Kaneko K , Ito T . Immunohistochemical study of proliferating cell nuclear antigen in hepatocytes of biliary atresia: a parameter to predict clinical outcome. J Pediatr Surg 1995; 30: 1297–1301. 8523229 10.1016/0022-3468(95)90489-1

[8] Gupta L , Gupta SD , Bhatnagar V . Extrahepatic biliary atresia: correlation of histopathology and liver function tests with surgical outcomes. J Indian Assoc Pediatr Surg 2012; 17: 147–152. 23243365 10.4103/0971-9261.102326 PMC3518991

[9] Tan CE , Davenport M , Driver M , Howard ER . Does the morphology of the extrahepatic biliary remnants in biliary atresia influence survival? A review of 205 cases. J Pediatr Surg 1994; 29: 1459–1464. 7844721 10.1016/0022-3468(94)90144-9

[10] Azarow KS , Phillips MJ , Sandler AD , Hagerstrand I , Superina RA . Biliary atresia: should all patients undergo a portoenterostomy? J Pediatr Surg 1997; 32: 168–174. 9044116 10.1016/s0022-3468(97)90173-1

[11] Superina R. Biliary atresia and liver transplantation: results and thoughts for primary liver transplantation in select patients. Pediatr Surg Int 2017; 33: 1297–1304. 29030698 10.1007/s00383-017-4174-4

[12] Weerasooriya VS , White FV , Shepherd RW . Hepatic fibrosis and survival in biliary atresia. J Pediatr 2004; 144: 123–125. 14722530 10.1016/j.jpeds.2003.09.042

[13] Santos JL , Kieling CO , Meurer L , Vieira S , Ferreira CT , Lorentz A *et al* The extent of biliary proliferation in liver biopsies from patients with biliary atresia at portoenterostomy is associated with the postoperative prognosis. J Pediatr Surg 2009; 44: 695–701. 19361628 10.1016/j.jpedsurg.2008.09.013

[14] Roy P , Chatterjee U , Ganguli M , Banerjee S , Chatterjee SK , Basu AK . A histopathological study of liver and biliary remnants with clinical outcome in cases of extrahepatic biliary atresia. Indian J Pathol Microbiol 2010; 53: 101–105. 20090233 10.4103/0377-4929.59194

[15] Lopez RN , Ooi CY , Krishnan U . Early and peri‐operative prognostic indicators in infants undergoing hepatic portoenterostomy for biliary atresia: a review. Curr Gastroenterol Rep 2017; 19: 16. 28374309 10.1007/s11894-017-0555-z

[16] Penault‐Llorca F , Radosevic‐Robin N . Ki67 assessment in breast cancer: an update. Pathology 2017; 49: 166–171. 28065411 10.1016/j.pathol.2016.11.006

[17] Ojanguren I , Ariza A , Llatjos M , Castella E , Mate JL , Navas‐Palacios JJ . Proliferating cell nuclear antigen expression in normal, regenerative, and neoplastic liver: a fine‐needle aspiration cytology and biopsy study. Hum Pathol 1993; 24: 905–908. 7690737 10.1016/0046-8177(93)90141-3

[18] Farinati F , Cardin R , D'Errico A , De Maria N , Naccarato R , Cecchetto A *et al* Hepatocyte proliferative activity in chronic liver damage as assessed by the monoclonal antibody MIB1 Ki67 in archival material: the role of etiology, disease activity, iron, and lipid peroxidation. Hepatology 1996; 23: 1468–1475. 8675166 10.1053/jhep.1996.v23.pm0008675166

[19] Marongiu F , Serra MP , Sini M , Marongiu M , Contini A , Laconi E . Cell turnover in the repopulated rat liver: distinct lineages for hepatocytes and the biliary epithelium. Cell Tissue Res 2014; 356: 333–340. 24687306 10.1007/s00441-014-1800-5 PMC4015059

[20] Karidis NP , Delladetsima I , Theocharis S . Hepatocyte turnover in chronic HCV‐induced liver injury and cirrhosis. Gastroenterol Res Pract 2015; 2015: 654105. 25892989 10.1155/2015/654105 PMC4393903

[21] Kinugasa Y , Nakashima Y , Matsuo S , Shono K , Suita S , Sueishi K . Bile ductular proliferation as a prognostic factor in biliary atresia: an immunohistochemical assessment. J Pediatr Surg 1999; 34: 1715–1720. 10591578 10.1016/s0022-3468(99)90652-8

[22] Ichida F , Tsuji T , Omata M , Ichida T , Inoue K , Kamimura T *et al* New Inuyama classification; new criteria for histological assessment of chronic hepatitis. Int Hepatol Commun 1996; 6: 112–119.

[23] Nakagawa T , Ohnishi K , Kosaki Y , Saito Y , Horlad H , Fujiwara Y *et al* Optimum immunohistochemical procedures for analysis of macrophages in human and mouse formalin fixed paraffin‐embedded tissue samples. J Clin Exp Hematop 2017; 57: 31–36. 28679964 10.3960/jslrt.17017 PMC6144271

[24] Funaki N , Sasano H , Shizawa S , Nio M , Iwami D , Ohi R *et al* Apoptosis and cell proliferation in biliary atresia. J Pathol 1998; 186: 429–433. 10209494 10.1002/(SICI)1096-9896(199812)186:4<429::AID-PATH195>3.0.CO;2-6

[25] Raap M , Liessem S , Ruschoff J , Fisseler‐Eckhoff A , Reiner A , Dirnhofer S *et al* Quality assurance trials for Ki67 assessment in pathology. Virchows Arch 2017; 471: 501–508. 28497316 10.1007/s00428-017-2142-y

[26] Longo‐Santos LR , Teodoro WR , de Mello ES , Velosa AP , Parra ER , Capelozzi VL *et al* Early type I collagen deposition is associated with prognosis in biliary atresia. J Pediatr Surg 2016; 51: 379–385. 26452701 10.1016/j.jpedsurg.2015.08.061

[27] Shteyer E , Ramm GA , Xu C , White FV , Shepherd RW . Outcome after portoenterostomy in biliary atresia: pivotal role of degree of liver fibrosis and intensity of stellate cell activation. J Pediatr Gastroenterol Nutr 2006; 42: 93–99. 16385261 10.1097/01.mpg.0000189324.80323.a6

[28] Suominen JS , Lampela H , Heikkila P , Lohi J , Jalanko H , Pakarinen MP . Myofibroblastic cell activation and neovascularization predict native liver survival and development of esophageal varices in biliary atresia. World J Gastroenterol 2014; 20: 3312–3319. 24696612 10.3748/wjg.v20.i12.3312 PMC3964401

[29] Dong R , Luo Y , Zheng S . Alpha‐SMA overexpression associated with increased liver fibrosis in infants with biliary atresia. J Pediatr Gastroenterol Nutr 2012; 55: 653–656. 22785417 10.1097/MPG.0b013e3182680be3

[30] Kazankov K , Barrera F , Moller HJ , Bibby BM , Vilstrup H , George J *et al* Soluble CD163, a macrophage activation marker, is independently associated with fibrosis in patients with chronic viral hepatitis B and C. Hepatology 2014; 60: 521–530. 24623375 10.1002/hep.27129

[31] Mantovani A , Biswas SK , Galdiero MR , Sica A , Locati M . Macrophage plasticity and polarization in tissue repair and remodelling. J Pathol 2013; 229: 176–185. 23096265 10.1002/path.4133

[32] Lertudomphonwanit C , Mourya R , Fei L , Zhang Y , Gutta S , Yang L *et al* Large‐scale proteomics identifies MMP‐7 as a sentinel of epithelial injury and of biliary atresia. *Sci Transl Med* 2017; **9**: eaan8462. 10.1126/scitranslmed.aan8462 PMC5902315 29167395

[33] Parks WC , Wilson CL , Lopez‐Boado YS . Matrix metalloproteinases as modulators of inflammation and innate immunity. Nat Rev Immunol 2004; 4: 617–629. 15286728 10.1038/nri1418

[34] Nio M. Japanese Biliary Atresia Registry. Pediatr Surg Int 2017; 33: 1319–1325. 29039049 10.1007/s00383-017-4160-x

