# Supplementary material for: Ki67 expression at Kasai portoenterostomy as a prognostic factor in patients with biliary atresia
Source: BJS Open. 2020 Jun 16;4(5):873–83. doi: 10.1002/bjs5.50308 (PMC7528526; doi:10.1002/bjs5.50308)
Supplement: Supplementary file 1 — Appendix S1: Supporting information [file BJS5-4-873-s001.docx]

**BJS5_50308**

**Ki67 expression at Kasai portoenterostomy as a prognostic factor in patients with biliary atresia**

**D. Yoshii, Y. Inomata, Y. Komohara, K. Shimata, M. Honda, S. Hayashida, Y. Oya, H. Yamamoto, H. Yamamoto, Y. Sugawara and T. Hibi**

**Fig. S1** Representative receiver operating characteristic (ROC) curves for factors predictive of native liver survival in patients with biliary atresia after Kasai portoenterostomy


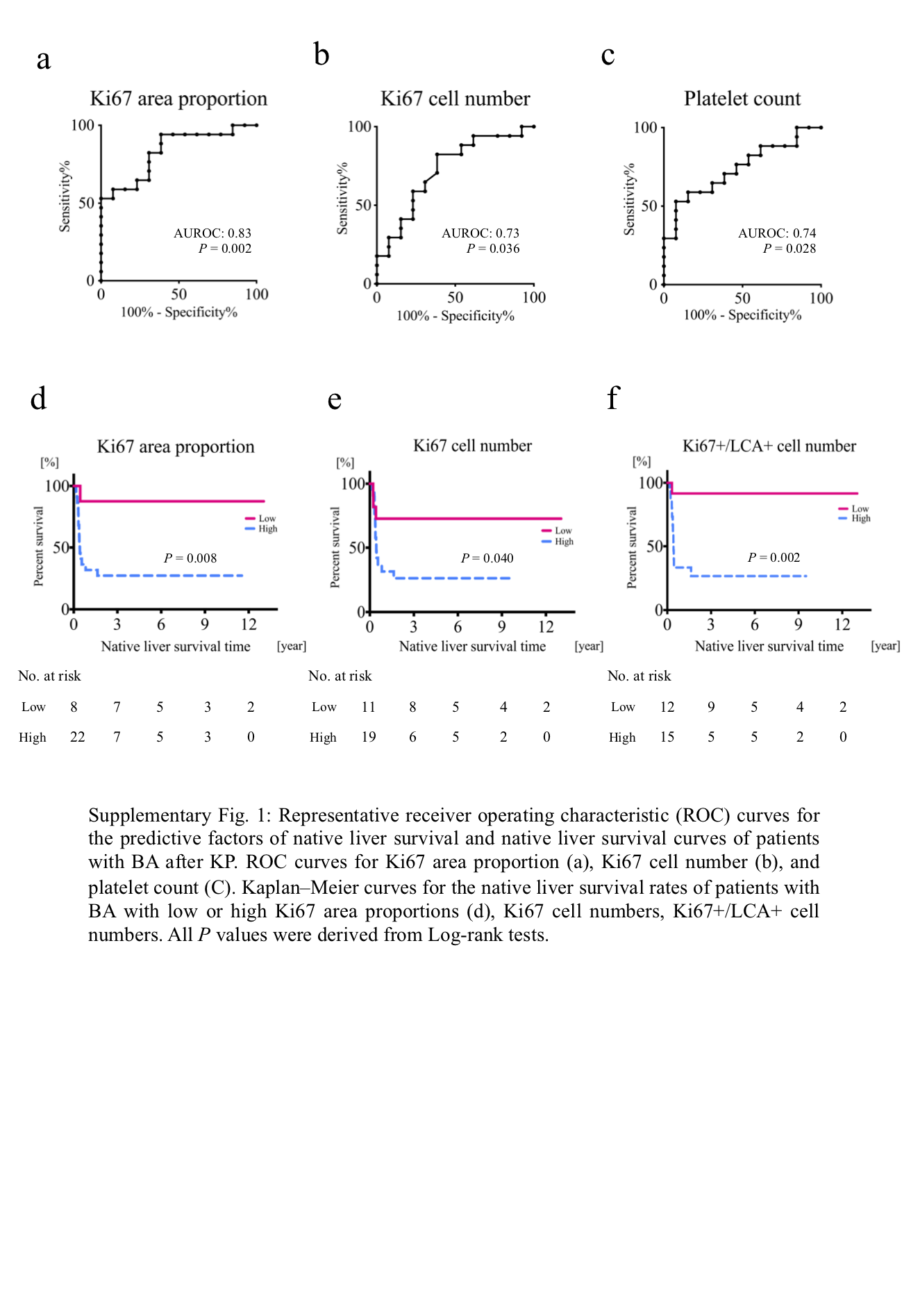


**Fig. S2** Associations between CD163 and Ki67 in liver specimens
